# First application of whole blood exchange–lymphoplasmapheresis combined transfusion for restoring immune homeostasis of ceftriaxone-induced hemolytic crisis: a case report

**DOI:** 10.3389/fimmu.2026.1821127

**Published:** 2026-07-09

**Authors:** Na Shen, Mu Zhang, Jingwei Li, Hongyan Chen, Yan Xing, Wenjun Que, Xiaoliang Yang

**Affiliations:** 1 Department of Critical Care Medicine, The First Affiliated Hospital of Chongqing Medical University, Chongqing, China; 2 Department of Blood Transfusion, Sichuan Provincial People’s Hospital, University of Electronic Science and Technology of China, Chengdu, China; 3 Department of Medical Laboratory Science, Yunyang County Maternal and Child Health Hospital, Chongqing, China; 4 Department of Blood Transfusion, The First Affiliated Hospital of Chongqing Medical University, Chongqing, China; 5 Department of Blood Transfusion, The Liangjiang Hospital of Chongqing Medical University, Chongqing, China

**Keywords:** ceftriaxone, drug-induced immune hemolytic anemia, hemolytic crisis, lymphoplasmapheresis, whole blood exchange

## Abstract

**Background:**

Ceftriaxone-induced hemolytic crisis is a rare, rapid, and severe drug adverse reaction. Once diagnosed, immediate discontinuation of ceftriaxone is the preferred treatment, but death remains unavoidable in some severe cases. This study reports a case of novel plasma exchange treatment for ceftriaxone-induced hemolytic crisis.

**Case presentation:**

A 57-year-old woman with a history of “acute gastroenteritis” still experienced abdominal pain after 4 days of treatment with “cefotaxime” in another hospital. One hour after receiving ceftriaxone in our hospital, the patient suddenly experienced dark brown urine and shock. Moreover, the hemoglobin (Hb) level declined sharply to 26 g/L with a positive direct Coombs test. The subsequent results indicated ceftriaxone antibody titers with IgG (1:2) and IgM (1:16) in the patient’s plasma, and the diagnosis was confirmed as ceftriaxone-induced hemolytic crisis. Immediately, ceftriaxone was discontinued. After anti-shock treatment, administration of glucocorticoids and whole blood exchange–lymphoplasmapheresis combined transfusion (WLCT) was performed. The hemolysis, liver function, and IgM titer of the patient significantly improved, with the direct Coombs test being negative. The patient was discharged after 10 days of hospitalization and remained stable during the 3-month follow-up.

**Conclusion:**

This case was the first successful application of WLCT for ceftriaxone-induced acute hemolytic crisis. Moreover, WLCT may represent a promising therapeutic option for the rapid restoration of intravascular immune homeostasis, but its efficacy and safety remain to be validated in future studies.

## Introduction

Drug-induced immune hemolytic anemia (DIIHA) is a severe and rare adverse reaction when using the antibiotics cefotetan, ceftriaxone, and piperacillin/tazobactam (1, 2). Ceftriaxone-induced DIIHA is characterized by a rapid decline in hemoglobin, a positive direct Coombs test (DAT), organ failure, and even death. After identification, immediate drug withdrawal and implementation of a series of treatment measures (anti-shock, transfusion, and organ support) are performed, but death remains unavoidable in some severe cases (3, 4). Therefore, timely identification and treatment strategies can significantly improve the prognosis. Once diagnosed, immediate discontinuation of ceftriaxone is the preferred treatment (5, 6), and the subsequent restoration of immune homeostasis is also another very important measure for severe cases.

## Case presentation

**Figure 1:**
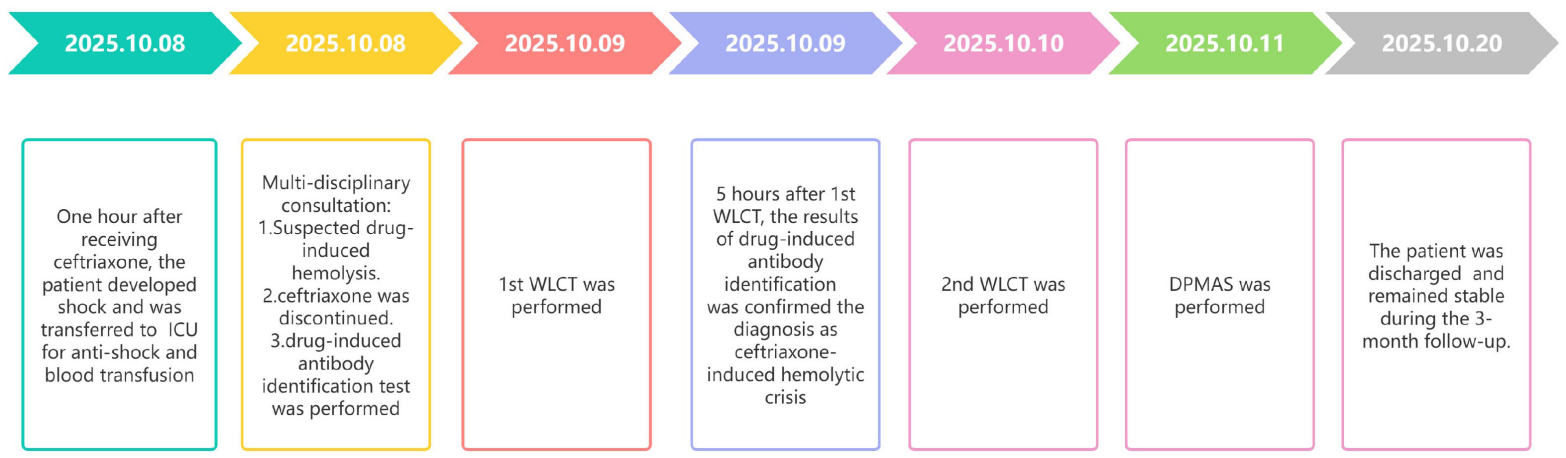
Clinical timeline schematic figure of whole blood exchange–lymphoplasmapheresis combined transfusion (WLCT) in treating ceftriaxone-induced immune hemolytic crisis. DPMAS, dual plasma molecular adsorption system.

**Figure 2:**
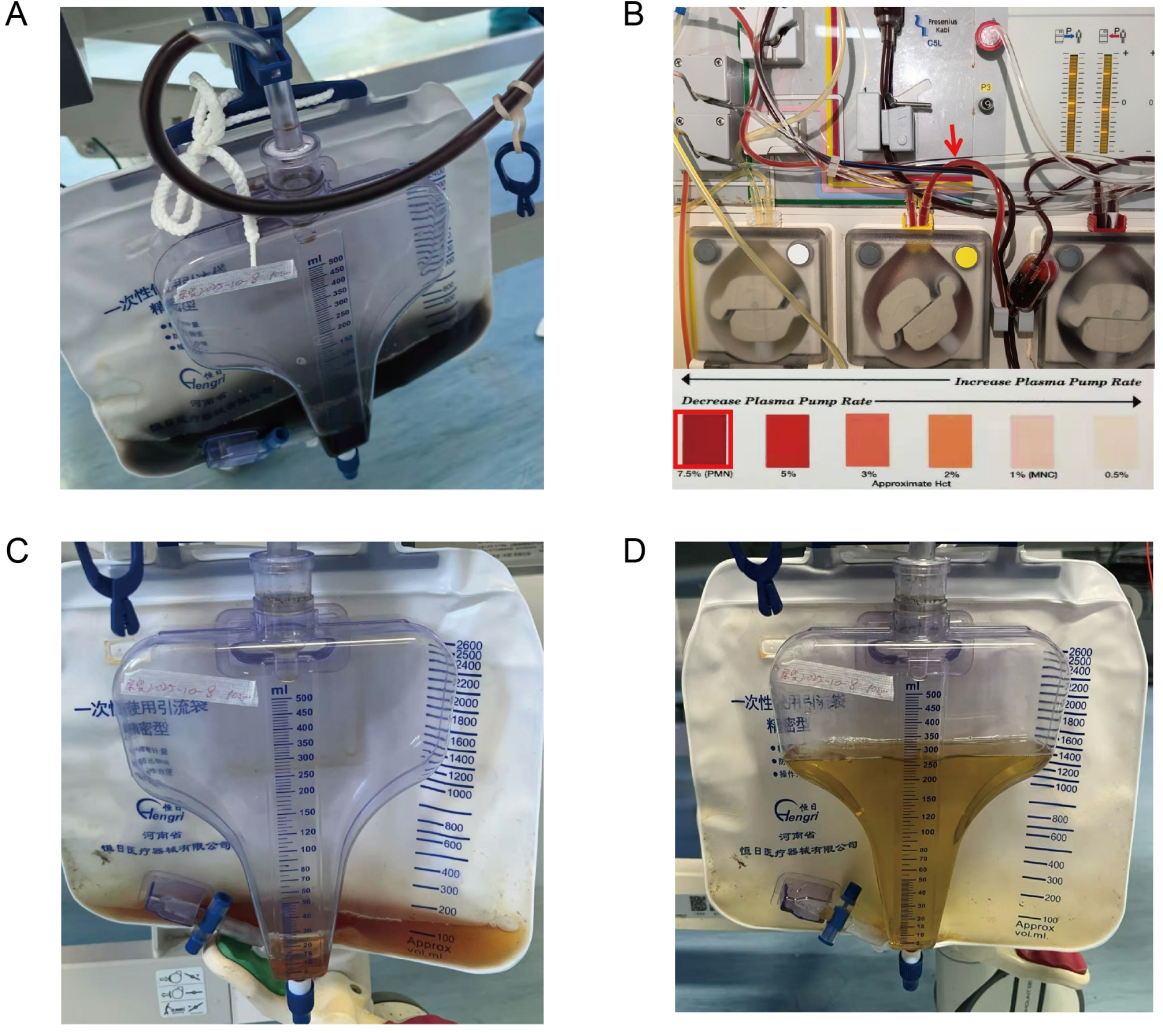
Clinical manifestations of whole blood exchange–lymphoplasmapheresis combined transfusion (WLCT) in treating ceftriaxone-induced immune hemolytic crisis. **(A)** Dark brown urine of patient before WLCT at intensive care unit (ICU) admission. **(B)** The color of pipeline from patient’s plasma collection bag (red arrow) matched the polymorphonuclear leukocyte (PMN; 7.5%) sample in the color chart (red rectangle). **(C)** The urine color after the first WLCT. **(D)** The urine color after the second WLCT.

The brief description of the entire process of this case report can be found in **Figure 1**. A 57-year-old woman with a history of “acute gastroenteritis” still experienced abdominal pain after 4 days of anti-infection treatment with “cefotaxime” in another hospital. On October 8, 2025, the patient received ceftriaxone for anti-infection and tramadol for pain relief in the emergency department of our hospital. One hour later, the patient suddenly experienced worsening abdominal pain, lethargy, and dark brown urine (**Figure 2A**). Meanwhile, the vital signs of the patient showed a series of deterioration, including heart rate (143 bpm), blood pressure (93/58 mmHg), and lactate level (12.2 mmol/L), and the limbs displayed marbled patterns. The patient was urgently transferred to the intensive care unit (ICU) due to shock, and the subsequent blood gas analysis showed that the hemoglobin (Hb) level was only 26 g/L. After anti-shock treatment and transfusion in ICU, follow-up laboratory indicators displayed the following: Hb (48 g/L), total bilirubin (260.3 μmol/L), direct bilirubin (203 μmol/L), indirect bilirubin (57.3 μmol/L), alanine aminotransferase (ALT; 3, 496 U/L), aspartate aminotransferase (AST; 3, 750 U/L), lactate dehydrogenase (LDH; 5, 000 U/L), and DAT (anti-IgG plus anti-C3d ++++, anti-IgG ++, and anti-C3d +++).

Following urgent admission to the ICU, multi-disciplinary consultation was initiated promptly to confirm the diagnosis. First, based on the patient’s past medical history and present signs, acute hemolysis was confirmed. Clinical manifestations, including significant bleeding, severe infection, and multiple organ dysfunction syndrome, were absent. Therefore, autoimmune hemolytic anemia, sepsis-associated hemolysis, Disseminated Intravascular Coagulation (DIC), thrombotic microangiopathy(TMA), and G6PD deficiency could be excluded. Second, the patient only received cefotaxime, tramadol, and ceftriaxone. There is no established immune mechanism or reported cases of acute, severe, IgM-mediated intravascular hemolysis caused by tramadol. Moreover, the patient had received cefotaxime for four consecutive days at another hospital without any signs or symptoms of hemolysis. Hemolysis occurred only after ceftriaxone administration in our hospital. Thus, ceftriaxone-induced immune hemolytic crisis was highly suspected.

After the multi-disciplinary consultation, a series of treatment measures were carried out simultaneously. First, ceftriaxone was discontinued and switched to imipenem and cilastatin immediately. Second, the drug-induced antibody identification test was performed to confirm the diagnosis, using methods outlined in a previous study (7). Meanwhile, based on the pathogenic mechanism, this patient required simultaneous and prompt removal of the three core elements [ceftriaxone-specific IgM antibodies, antibody-secreting plasma cells, and sensitized red blood cells (RBCs)] to restore the patient’s immune homeostasis rapidly. Moreover, we had to deal with the shortage of blood supply. Therefore, for this patient, the administration of glucocorticoids combined with whole blood exchange–lymphoplasmapheresis combined transfusion (WLCT) was the chosen therapy.

Employing the plasma exchange program and consumables (PL1) of the blood component separator (COM.TEC, Fresenius Kabi), WLCT was achieved by turning off the interface position control and reducing the plasma flow rate gradually in a manual way. When the color of the pipeline from the patient’s plasma collection bag was a little darker than that of the polymorphonuclear leukocyte (PMN; 7.5%) sample in the color chart (**Figure 2B**), the operation remained stable. Although this innovative operation was not described in the instructions of the separator, our domestic patents of WLCT had already been granted (8). The scheme of WLCT on October 9, 2025, was performed at 0.6 times plasma volume with replacement fluids (RBC 4 U + 1, 000 mL plasma), which only took 55 min.

**Figure 3:**
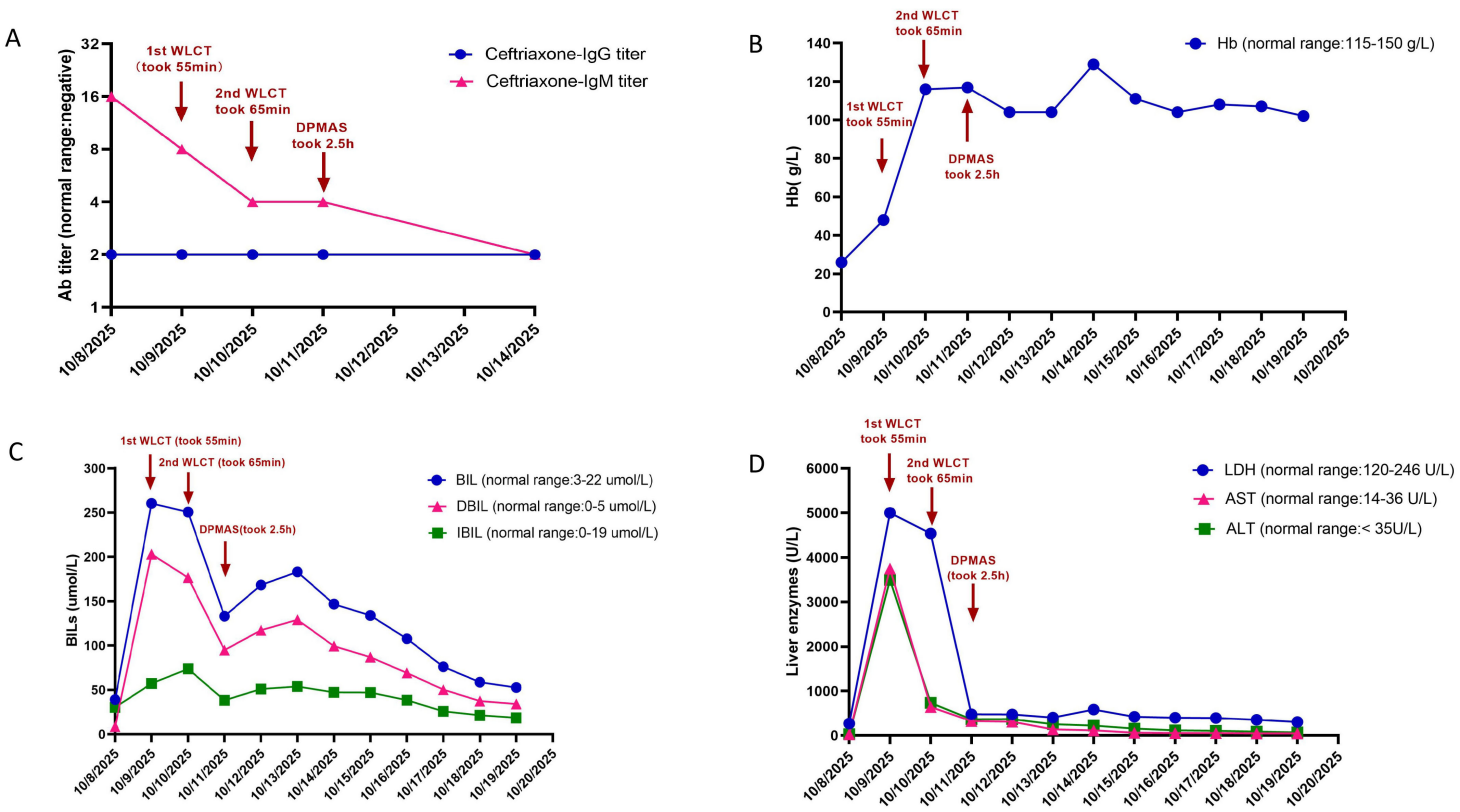
Laboratory indicators of whole blood exchange–lymphoplasmapheresis combined transfusion (WLCT) and dual plasma molecular adsorption system (DPMAS) in treating ceftriaxone-induced immune hemolytic crisis. **(A)** Ceftriaxone antibody titers (IgG and IgM) of the patient for different days after intensive care unit (ICU) admission. **(B)** Hemoglobin (Hb) levels of patients on different days after ICU admission. **(C)** Bilirubin (BIL), direct bilirubin (DBIL), and indirect bilirubin (IBIL) of the patient for different days after ICU admission. **(D)** Lactate dehydrogenase (LDH), aspartate transaminase (AST), and alanine transaminase (ALT) of the patient on different days after ICU admission.

Furthermore, after 5 hours, the subsequent results of drug-induced antibody identification indicated that ceftriaxone antibody titers with IgG (1:2) and IgM (1:16) were in the patient’s plasma. Thus, the diagnosis was confirmed as ceftriaxone-induced hemolytic crisis. To restore the patient’s immune homeostasis persistently, the second WLCT on October 10, 2025, was also performed at 0.6 times plasma volume with replacement fluids (RBC 2 U + 1, 000 mL plasma), which only took 65 min. The bilirubin level remained 250.5 µmol/L after the second WLCT. The dual plasma molecular adsorption system (DPMAS) procedure was performed on October 11, 2025, with replacement fluids (1, 200 mL plasma), which took 2.5 h. Through subsequent comprehensive treatment, the urine color, Hb levels, hemolysis, liver function, infection, and IgM titer of the patient significantly improved (**Table 1**; **Figures 2C, D**, **3A–D**), with the DAT being negative. The patient was discharged after 10 days of hospitalization and remained stable during the 3-month follow-up.

**Table 1:** The reference ranges for all laboratory parameters.

Parameter	Reference ranges
BIL (μmol/L)	3–22
DBIL (μmol/L)	0–5
IBIL (μmol/L)	0–19
Hb (g/L)	115–150
AST (U/L)	14–36
ALT (U/L)	<35
LDH (U/L)	120–246
DAT	Negative

BIL, bilirubin; DBIL, direct bilirubin; IBIL, indirect bilirubin; Hb, hemoglobin; AST, aspartate transaminase; ALT, alanine transaminase; LDH, lactate dehydrogenase; DAT, direct Coombs test.

## Discussion

Ceftriaxone is usually used in the treatment of infections in the lower respiratory tract, urinary tract, biliary tract, and others. Ceftriaxone-induced DIIHA is highly prone to misdiagnosis, with a morbidity of approximately one in a million people per year and a mortality rate of at least 30%. The clinical manifestations of ceftriaxone-induced DIIHA are usually more severe in children than in adults (6, 9). Due to the binding of ceftriaxone-specific IgM antibodies to the complex antigens on the surface of RBCs, the classical complement pathway can be rapidly activated, forming a membrane attack complex, leading to RBC destruction and hemoglobin release (manifested as dark brown urine); severe adverse reactions include anaphylactic shock, hemolysis, hematuria, and liver and kidney injury, among which acute immune hemolysis reaction is one of the more serious adverse reactions (9).

Although ceftriaxone-induced hemolytic crisis is prone to being missed, our emergency physicians still promptly identified it. Once diagnosed, drug withdrawal is the first and most important treatment measure to perform. In fact, the subsequent restoration of immune homeostasis is also another very important measure affecting the prognosis of patients with severe cases. Active removal of drug-induced antibodies by plasma exchange has been performed for DIIHA (9, 10). In severe cases, conventional drug withdrawal treatments could merely prevent the allergen from re-stimulating the host, but sensitized RBCs of the positive DAT remain, which could further increase the burden on hemolysis and renal failure. CD47, highly expressed on healthy RBCs, binds to SIRPα and transmits an inhibitory signal downstream, preventing macrophage activation and phagocytosis. This “don’t eat me” signal ensures that red blood cells can complete their average 120-day circulatory lifespan. Once RBCs are damaged, CD47 expression on RBCs decreases, or its binding capacity to SIRPα is weakened, which leads to an aggravation of hemolysis (11). In addition, over-activated immune cells continuously exist and produce antibodies, which can lead to the disruption of the stability of the immune internal environment (2, 6, 9). Thus, drug withdrawal was not sufficient to quickly restore the homeostasis of the internal environment, avoid multiple organ failure, and improve the patient’s condition in these severe cases. More rapid and effective treatment measures are urgently needed.

It is well known that there are currently three types of plasma exchange methods: therapeutic plasma exchange (TPE), lymphoplasmapheresis, and whole blood exchange. Conventional TPE can only relatively reduce the antibody titers in plasma (12, 13) but fails to eliminate antibody-secreting plasma cells and sensitized RBCs. Several studies have shown that compared with TPE, lymphoplasmapheresis achieved better prognosis through reduced plasma volume in multiple autoimmune diseases, e.g., myasthenia gravis, myasthenia crisis, steroid-refractory neuromyelitis optica spectrum disorder, and thrombotic thrombocytopenic purpura (14–17). Although this technique can remove plasma cells by adjusting the parameters of the plasma exchange procedure and reduce the antibody titers simultaneously, it cannot eliminate sensitized red blood cells.

Whole blood exchange is a treatment method to improve the internal environment of the patient rapidly for some special critical patients (such as transfusion reactions due to blood type incompatibility and poisoning), especially achieving remarkable results in severe autoimmune hemolytic anemia (18–21). The achievement has been incorporated into the seventh special issue of the American Society for Apheresis (ASFA) guidelines (22). This technique can relatively reduce the antibody titers and eliminate antibody-secreting plasma cells and sensitized RBCs, but the current situation of blood shortage in China cannot meet the requirement of a large amount of RBCs for whole blood exchange (23).

The reasons for DPMAS being introduced after the second WLCT rather than earlier were as follows: first, based on the pathogenic mechanism, this patient required simultaneous and prompt removal of the three core elements to restore the patient’s immune homeostasis rapidly and persistently. DPMAS can only relatively reduce bilirubin and the antibody titers in plasma, but fails to eliminate antibody-secreting plasma cells and sensitized RBCs. WLCT could remove the three core elements simultaneously to restore the patient’s immune homeostasis rapidly. Second, after the second WLCT, the patient’s condition stabilized, and primary laboratory findings improved, but bilirubin was still at a high level. Accordingly, DPMAS was initiated to reduce bilirubin concentrations. Third, the cost of DPMAS was approximately RMB 11, 000 per session, which was quite higher than that of WLCT (RMB 5, 000 per session). Moreover, if the bilirubin level is elevated persistently after WLCT, simultaneous or alternating use of WLCT and DPMAS could be a better choice.

In contrast, based on lymphoplasmapheresis and whole blood exchange, WLCT is a novel and improved plasma exchange designed to provide targeted intervention against disease etiology, which can overcome the shortcomings of the aforementioned methods by reducing the antibody titers in plasma and removing antibody-secreting plasma cells and sensitized RBCs with less RBC volume simultaneously. Our novel method is characterized by a lower exchange volume, shorter exchange time, lower amount of RBCs, and less treatment frequency, which are crucial for the rapid restoration of intravascular immune homeostasis.

Based on the pathogenic mechanism, the treatment plan for this patient should eliminate the three core elements (ceftriaxone-specific IgM antibodies, antibody-secreting plasma cells, and sensitized RBCs) simultaneously as soon as possible to restore the patient’s immune homeostasis. Meanwhile, we also had to deal with the shortage of blood supply. Therefore, for this patient, WLCT combined with glucocorticoids was chosen as the therapy plan, not plasma exchange. Finally, it is necessary to emphasize that the improvement of this patient was the result of comprehensive multidisciplinary intensive care, not attributable solely to WLCT. Despite the findings, the mechanism of WLCT in treating hemolytic crisis remains unexplored. Further multi-center prospective studies should be performed to validate the problem and provide a deeper understanding of this novel technique.

## Conclusion

This case was the first successful application of WLCT for ceftriaxone-induced acute immune hemolytic crisis, demonstrating the clinical utility of this technique in the management of DIIHA. Moreover, WLCT may represent a promising therapeutic option for the rapid restoration of intravascular immune homeostasis, but its efficacy and safety remain to be validated in future studies.

## Data Availability

The original contributions presented in the study are included in the article/supplementary material. Further inquiries can be directed to the corresponding author.
